# Synergistic role of blood-labyrinth barrier permeability and endolymphatic hydrops: a comparative perspective in Ménière’s disease and vestibular migraine

**DOI:** 10.3389/fneur.2025.1667277

**Published:** 2025-09-03

**Authors:** Haozhe Yin, Hui Li, Yifan Zheng, Yanlu Jia, Bo Shen, Yuanyuan Sun, Shuning Sun, Yaoheng Zhang, Wenbo Peng, Chunling Liu

**Affiliations:** ^1^Department of Neurology, The Second Affiliated Hospital of Zhengzhou University, Zhengzhou, China; ^2^Department of Neurology, The First Affiliated Hospital of Jiamusi University, Jiamusi, China

**Keywords:** Ménière’s disease, vestibular migraine, endolymphatic hydrops, blood-labyrinth barrier, hearing loss

## Abstract

**Objective:**

This study compares the delayed gadolinium-enhanced MRI characteristics of Ménière’s disease (MD) and vestibular migraine (VM) to develop a multiparametric model that incorporates endolymphatic hydrops (EH), blood-labyrinth barrier permeability, and their asymmetry. Additionally, it investigates the correlations between these imaging features and hearing loss across various frequencies.

**Methods:**

A total of 79 patients—47 with MD and 32 with VM—were enrolled in the study between June 2023 and April 2025. All participants underwent a comprehensive medical history assessment, neurotologic evaluation, audiological testing, and a 3D SPACE FLAIR MRI conducted 4 h after the administration of intravenous gadolinium contrast agents. EH, the signal intensity ratio (SIR) of the cochlear basal turn, and the cochlear SIR asymmetry index (c-SIR AI) were assessed. Linear regression was employed to evaluate the contributions of EH and SIR to hearing loss. Additionally, a logistic regression model with ROC analysis was developed for diagnostic purposes.

**Results:**

All ipsilateral ears in the MD group exhibited EH, with median cochlear and vestibular EH grades of 2 (1, 2) and 1 (1, 2), respectively. These grades were significantly higher than those observed in the VM group, where the median grades were 0 (0, 1) for both cochlear and vestibular EH (both *p* < 0.001). MD demonstrated a unilateral predominance. The ipsilateral SIR and c-SIR AI were higher in MD compared to VM (1.39 ± 0.15 vs. 1.18 ± 0.18 and 17.24 ± 10.93 vs. 6.52 ± 3.74, both *p* < 0.001). In MD, both EH and SIR predicted low-frequency hearing loss; with SIR being the primary predictor (*β* = 68.717, *p* < 0.001). SIR also predicted high-frequency loss (β = 80.139, p < 0.001). In VM, SIR predicted thresholds across all frequencies, with the strongest correlation observed for high-frequency thresholds (*β* = 79.551, *p* < 0.001). A combined model (including cochlear EH, vestibular EH, SIR, and c-SIR AI) demonstrated high diagnostic performance, achieving a sensitivity of 76.6%, specificity of 100%, and an AUC of 0.954.

**Conclusion:**

Delayed gadolinium-enhanced MRI of the inner ear facilitates the differentiation between MD and VM. The combination of EH, SIR, and c-SIR AI demonstrates excellent diagnostic performance. Notably, elevated SIR shows the strongest correlation with high-frequency hearing impairment, while cochlear EH primarily contributes to low- and mid-frequency hearing loss. By integrating imaging findings with audiological profiles, clinicians can accurately characterize cochlear pathology, enabling the development of tailored treatment strategies.

## 1 Introduction

Ménière’s disease (MD) and vestibular migraine (VM) are two significant causes of episodic vestibular syndrome. MD is primarily characterized by recurrent vertigo attacks, fluctuating hearing loss, tinnitus, and aural fullness (1), while VM is defined by the coexistence of migraine (with or without aura) and episodic vestibular symptoms (2). However, patient self-reports and the dynamic nature of symptoms significantly affect diagnostic accuracy. The overlapping and comorbid presentations of MD and VM present substantial challenges to differential diagnosis. Although fluctuating hearing loss is a hallmark of MD, a subset of VM patients may also exhibit mild to moderate hearing impairment. Vestibular function tests, such as caloric testing and video head impulse testing (vHIT), provide limited diagnostic utility in differentiating MD from VM and are more commonly used to assess disease stage and the extent of vestibular dysfunction (3). In summary, the combination of symptom-based and functional assessments (vestibular and audiological) may facilitate the diagnosis of typical MD or VM; however, they remain insufficient for patients with overlapping or atypical presentations.

Faced with the “gray zone” between symptomatology and functional testing, delayed gadolinium-enhanced MRI of the inner ear—capable of visualizing endolymphatic hydrops (EH)—has emerged as a pivotal tool for resolving diagnostic challenges (4). EH is commonly regarded as the morphological pathological basis of MD (5), and its severity and distribution pattern (involving the cochlea and/or vestibule) are closely associated with the clinical manifestations of MD (6–10). However, the causal relationship between EH and MD remains incompletely understood. EH can also be observed in asymptomatic individuals and patients with VM, suggesting that EH is not the sole pathophysiological driver of MD (11–13). The pathological significance of EH in both MD and VM warrants further investigation (14–16). Repeated inner ear ischemia in VM may underlie the tendency toward bilateral EH observed in VM patients (16), which could partly explain the slowly progressive and symmetrical hearing loss seen in some cases (17). While fluctuating low-frequency hearing loss is a hallmark of MD and is thought to be closely related to EH, the patterns of hearing loss (low-, mid-, or high-frequency) and their severity vary widely across individuals (6, 18).

Perilymphatic enhancement (PE) observed on delayed gadolinium-enhanced MRI of the inner ear reflects altered permeability of the blood-labyrinth barrier (BLB), representing another potential pathophysiological mechanism of MD. Increased BLB permeability can be induced by various factors including diuretics, inflammation, acoustic trauma, and hypoxia (18–22). The coexistence of EH and PE is a notable imaging feature of MD (19). However, visually comparing the ipsilateral and contralateral ears to assess the presence of PE may overlook subtle changes (15, 19, 23, 24). The signal intensity ratio (SIR) measured at the cochlear basal turn provides a quantitative biomarker for assessing BLB permeability. This metric offers insights into MD symptomatology and further elucidates its underlying pathophysiology. Clinical studies establish that SIR values in MD patients demonstrate significant positive correlations with advancing age, prolonged tinnitus duration, and elevated hearing thresholds in ipsilateral ears (9). Furthermore, studies have reported that the SIR of the contralateral ear in MD patients is higher than that in healthy individuals and those with sudden sensorineural hearing loss (20, 22), suggesting a potential systemic component in MD. We speculate that subtle PE, not readily detectable by visual inspection, may have been underestimated. Quantifying bilateral BLB permeability (SIR) and cochlear SIR asymmetry index (c-SIR AI) may help address this issue.

Relying solely on EH for differentiating between MD and VM presents significant limitations. This study aims to systematically compare patients with MD and VM regarding morphological alterations (EH) and metabolic dysfunction (as reflected by blood-labyrinth barrier permeability), using quantitative metrics including signal intensity ratio, cochlear SIR asymmetry index, cochlear endolymphatic hydrops (cEH), and vestibular endolymphatic hydrops (vEH). Furthermore, this study investigates how these imaging features influence hearing outcomes, thereby providing a novel perspective on the pathophysiological distinctions between the two disorders.

## 2 Methods

### 2.1 Patients

This study employed a cross-sectional case–control design, focusing on patients who presented with episodic vestibular syndrome at the Vertigo Center from June 2023 to April 2025. Following comprehensive history taking, all participants underwent pure-tone audiometry, neurotologic evaluation, and delayed gadolinium-enhanced MRI of the inner ear. The duration of disease was defined as the interval from the onset of the first vertigo episode to the time of evaluation date. “MD-ear symptoms” was defined as the following auditory symptoms: aural pressure, tinnitus, and self-reported hearing loss, which are characteristic of MD. The following exclusion criteria were applied: (1) patients who met only the criteria for probable MD or probable VM; (2) episodic vestibular syndrome secondary to external or middle ear disorders (e.g., otitis media) or structural inner ear malformations (e.g., enlarged vestibular aqueduct syndrome or congenital cochlear anomalies); (3) confirmed central causes of vertigo, such as transient ischemic attack (TIA), autoimmune encephalitis, or other severe psychiatric disorders; (4) hepatic or renal insufficiency; (5) patients who fulfilled the diagnostic criteria for both VM and MD simultaneously; (6) incomplete clinical data. Ultimately, the study included 47 patients with a clinical diagnosis of definite MD, as defined by the 2015 diagnostic criteria of the Bárány Society (1) and 32 patients with definite VM, according to the 2022 consensus criteria established by the Bárány Society and the International Headache Society (2). Written informed consent was obtained from all participants and the study received approval from the Ethics Committee of the Second Affiliated Hospital of Zhengzhou University (approval number: KY2025142).

### 2.2 Pure tone audiometry test

All patients underwent pure-tone audiometry to determine air and bone conduction thresholds before undergoing delayed gadolinium-enhanced MRI of the inner ear. The pure tone average (PTA) was calculated as the mean hearing threshold at frequencies of 500 Hz, 1 kHz, 2 kHz, and 4 kHz. Low-frequency thresholds were defined as the average of 250 Hz and 500 Hz, mid-frequency thresholds as the average of 1 kHz and 2 kHz, and high-frequency thresholds as the average of 4 kHz and 8 kHz, respectively.

### 2.3 Neurotologic evaluation

Neurotologic evaluation comprised videonystagmography (VNG) and the video head impulse test (vHIT), both employed to exclude central causes of vertigo and to assess peripheral vestibular function. As part of the VNG, the caloric test was conducted, and canal paresis (CP%) was calculated to evaluate unilateral vestibular hypofunction. A C*p* value exceeding 25% was deemed indicative of reduced function in the ipsilateral vestibular system. The vHIT was utilized to assess the vestibulo-ocular reflex (VOR) and to evaluate the functionality of the semicircular canal system. This test involved monitoring eye movements during rapid, passive, low-amplitude head impulses to detect compensatory saccades or changes in VOR gain. Semicircular canal dysfunction was identified by reduced gain (horizontal canal gain < 0.8; anterior/posterior canal gain < 0.7) accompanied by overt or covert catch-up saccades. In this study, we reported the overall abnormality rates of both caloric and head impulse tests.

### 2.4 Image

All patients underwent MRI using 3 T scanners equipped with a 20-channel head and neck coil, 4 h after the intravenous injection of a double dose of gadolinium contrast agents (0.2 mmol/kg). Prior to gadodiamide injection, a 3D SPACE T2 sequence was acquired for anatomical reference of the labyrinthine fluid space, with the following parameters: TE = 259 ms, TR = 1,200 ms, Matrix Size = 320 × 320, FOV = 190 mm × 190 mm, slice thickness = 0.4 mm, and acquisition time = 4 min and 28 s. The imaging protocol included a 3D SPACE FLAIR sequence with the following parameters: echo time (TE) = 373 ms, repetition time (TR) = 5,000 ms, inversion time (TI) = 1,650 ms, Matrix Size = 384 × 307, field of view (FOV) = 226 mm × 226 mm, slice thickness = 0.6 mm, and acquisition time of 11 min and 15 s.

### 2.5 Image analysis

**Figure 1:**
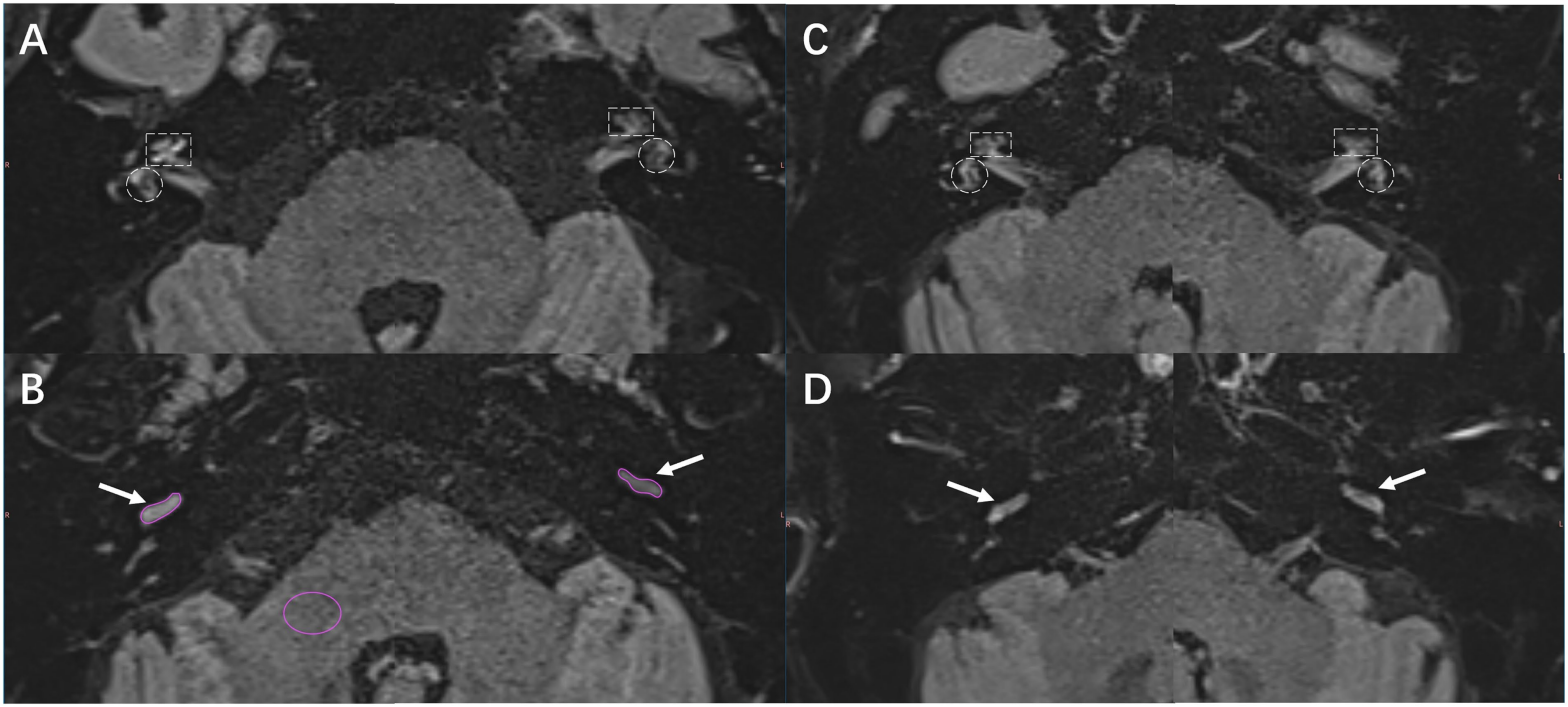
presents representative gadolinium-enhanced inner ear MRI images from patients diagnosed with Ménière’s disease (MD) and vestibular migraine (VM). **(A,B)** The images depict a 54-year-old male patient with MD. **(A)** Notable perilymph enhancement is observed in the cochlear basal turn of the right (ipsilateral) ear, characterized by grade 2 cEH and grade 2 vEH. In contrast, the left (contralateral) ear displays grade 1 cEH without vestibular hydrops. **(B)** The SIR measurement involved manually delineating ROIs on the cochlear basal turn and a 0.5 cm^2^ pontine area within the same slice. The SIR was computed as the ratio of mean cochlear signal intensity to mean pontine signal intensity. **(C,D)** The images of a 72-year-old female patient with VM are shown. **(C)** Bilateral grade 1 cEH is observed, with grade 1 vEH present on the left and no significant vEH on the right. **(D)** Symmetrical perilymph enhancement is noted in both cochlear basal turns. In all panels, the white dashed rectangles represent the cochlea, white dashed circles denote the vestibule, white arrows indicate the cochlear basal turn, and purple contours outline the ROIs for SIR measurement.

Signal intensity measurements and EH grading were independently conducted by two experienced neurotologists, Bo Shen and Yuanyuan Sun. The signal intensity ratio (SIR), which reflects the permeability of the blood-labyrinth barrier, was measured on the axial slice exhibiting the area of maximal enhancement in the cochlear basal turn (Figure 1B). Regions of interest (ROIs) were manually delineated over the cochlear basal turn ( $I_{\text{Cochlea}}$ ) and the pons ( $I_{\text{Pons}}\approx$ 0.5cm^2^) to calculate the SIR as:


$$\mathrm{SIR}=\frac{I_{\text{Cochlea}}}{I_{\text{Pons}}}$$

To quantify interaural differences in the SIR between ears in patients with VM and MD, the cochlear SIR asymmetry index (c-SIR AI) was defined as the ratio of the absolute difference between left and right cochlear SIR values to their average:


$$c-\mathrm{SIR}\;\mathrm{AI}=\frac{|{\mathrm{SIR}}_{L}-{\mathrm{SIR}}_{R}|\times 2}{{\mathrm{SIR}}_{L}+{\mathrm{SIR}}_{R}}\times 100\%$$

Vestibular and cochlear endolymphatic hydrops were graded using the semi-quantitative method proposed by Bernaerts (24) and Baráth (25). In the cochlea, endolymphatic hydrops (EH) was classified into three grades: grade 0 (normal), grade I (partial dilation of the scala media leading to a nodular indentation of the scala vestibuli), and grade II (complete obliteration of the scala vestibuli due to the distended cochlear duct). Vestibular EH was evaluated using a modified four-stage system: grade 0 indicates a normal saccule-to-utricle size ratio; grade I signifies that the saccule is equal to or larger than the utricle without confluence; grade II denotes confluence of the saccule and utricle with a residual peripheral perilymphatic rim; and grade III represents complete obliteration of the perilymphatic space. In instances of disagreement between the two evaluators, the final endolymphatic hydrops grading was determined by consensus through discussion and the final signal intensity values were derived from the average measurements of the two observers.

### 2.6 The clinically leading side

In our study, the term “ipsilateral” refers to the ear exhibiting symptoms similar to MD, such as aural fullness, tinnitus, in patients diagnosed with either MD or VM. For patients presenting bilateral symptoms, the ear displaying more pronounced MD-like features—namely, aural fullness, tinnitus, or fluctuating hearing loss—was designated as the ipsilateral ear. Conversely, the contralateral ear was defined as the opposite side. In VM patients lacking any MD-like aural symptoms, a random number between 0 and 9 was generated for each subject. An even number indicated the left ear as ipsilateral, while an odd number indicated the right ear as ipsilateral (14, 16). Unless otherwise specified, analyses within the VM group were conducted based on the ipsilateral side as defined above.

### 2.7 Statistical analysis

All statistical analyses were performed using IBM SPSS Statistics version 25.0 (IBM Corp., Armonk, NY, United States). Normally distributed continuous variables are expressed as mean ± standard deviation, while non-normally distributed ordinal data (e.g., endolymphatic hydrops grading) are summarized as median (interquartile range). Specifically, endolymphatic hydrops grades are reported as median (Q1, Q3) to accurately reflect their discrete and rank-ordered nature. The significance level (*α*) was set at 0.05, with a two-tailed *p* value < 0.05 deemed statistically significant. For comparisons between groups (MD vs. VM), continuous variables with normal distribution and equal variances were analyzed using independent-samples t-tests. In cases where homogeneity of variance was not met, Welch’s t-test was employed. For non-normally distributed data, the Mann–Whitney U test was utilized. For paired comparisons (ipsilateral vs. contralateral ears in the MD group), paired t-tests were conducted for normally distributed variables, while Wilcoxon signed-rank tests were applied for non-normally distributed variables. Categorical variables were analyzed using the chi-square test or Fisher’s exact test when expected frequencies were <5. The McNemar test was used to compare the distribution of EH grades between ipsilateral and contralateral ears in the MD group, and the chi-square test was employed to compare EH grade distributions between the MD and VM groups.

To investigate the relationship between imaging features and functional outcomes, exploratory simple linear regression analyses were initially conducted based on theoretical assumptions. The primary independent variables included age, disease duration, SIR, cochlear endolymphatic hydrops (cEH), and vestibular endolymphatic hydrops (vEH). Separate models were constructed for the Meniere’s Disease (MD) and Vestibular Migraine (VM) groups to examine associations with low-, mid-, and high-frequency hearing thresholds, as well as the pure-tone average (PTA). Independent variables with a *p*-value of less than 0.05 in the simple linear regression analyses were subsequently included in multiple linear regression analyses to assess their independent contributions to the corresponding hearing outcomes.

## 3 Results

### 3.1 Descriptive characteristics of the patients

A total of 47 patients diagnosed with definite MD and 32 patients diagnosed with definite VM were included in the final analysis. No significant differences in age or disease duration were observed between the MD and VM groups (*p* > 0.05). Although the rate of abnormal caloric responses was higher in the MD group (42.6%) compared to the VM group (28.1%), this difference was not statistically significant (*p* = 0.182). The abnormal rate of vHIT results was low in both groups, with 19.2% in MD and 12.5% in VM, and no significant difference was found (*p* = 0.566). Among the 47 MD patients, 8 (17.0%) had a prior history of migraine but did not meet the diagnostic criteria for VM. Conversely, among the 32 VM patients, 4 (12.5%) reported MD-like aural symptoms but did not present with fluctuating hearing loss, thereby failing to fulfill the diagnostic criteria for MD. A significant difference was noted in the proportion of female patients between the two groups, with 75.0% in the VM group compared to 53.2% in the MD group (*p* = 0.05; see Table 1).

**Table 1:** Descriptive characteristics of the patients.

Items	MD (*n* = 47)	VM (*n* = 32)	*t*/Z/*χ*^2^-value	*p*-value
General characteristics				
Age (years)	49.45 ± 14.67	47.44 ± 15.43	0.580	0.564^a^
Female, *n* (%)	25 (53.2%)	24 (75.0%)	3.84	**0.05** ^b^
Duration (month)	38.0 (3.0,130.0)	27.5 (4.5,141.0)	−0.45	0.964^c^
History of migraine, *n* (%)	8 (17.0%)	32 (100.0%)	52.44	**0.000** ^b^
MD-ear symptoms, *n* (%)	47 (100.0%)	4 (12.5%)	63.70	**0.000** ^b^
Abnormal vestibualr function tests				
Caloric test, *n* (%)	20 (42.6%)	9 (28.1%)	1.782	0.182^b^
vHIT, *n* (%)	9 (19.1%)	4 (12.5%)	0.614	0.566^b^

^a^Independent samples *t*-test (if equal variance assumed) or Welch’s corrected *t*-test (if unequal variance confirmed by Levene’s test); ^b^Chi-square test (for expected cell frequencies ≥5) or Fisher’s exact test (for expected frequencies <5); ^c^Mann–Whitney U test; MD-ear symptoms, fluctuating sensorineural hearing loss, and ipsilateral auditory symptoms (tinnitus or aural fullness),vHIT, video head impulse test. Bold values represent significant differences (*p* ≤ 0.05, as determined by the Chi-square test).

### 3.2 Audiological and imaging characteristics of Ménière’s disease and vestibular migraine

This study found that hearing loss in the ipsilateral ears of patients with MD was significantly more severe than in their contralateral ears and in patients with VM. Specifically, the PTA, as well as the low-frequency, mid-frequency, and high-frequency thresholds in the ipsilateral ears of MD patients were recorded at 41.0 (33.8, 59.0), 45.0 (37.5, 52.5), 42.5 (27.5, 60.0), and 57.5 (47.5, 67.5) dB HL, respectively—all of which were significantly higher than those observed in the contralateral ears of MD patients and in VM patients (*p* < 0.001 for all comparisons).

**Figure 2:**
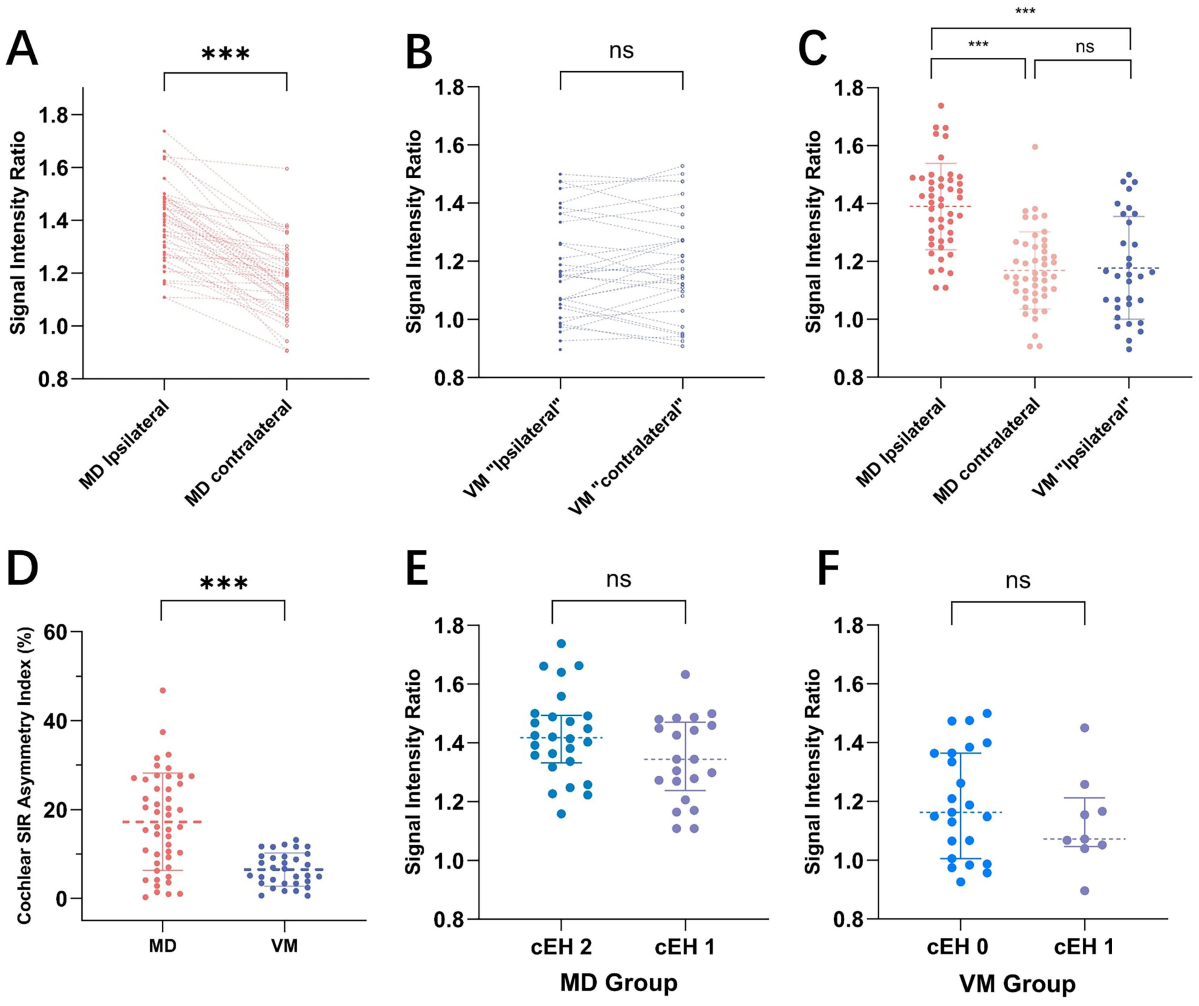
presents a comparison of the signal intensity ratio (SIR) between Meniere’s Disease (MD) and Vestibular Migraine (VM). Panels **A** and **B** illustrate paired comparisons of ipsilateral and contralateral ear SIRs for MD **(A)** and VM **(B)** patients, respectively. Each dotted line connects the SIR values of the ipsilateral and contralateral ears for individual subjects. A notable difference in SIR is observed between the sides in MD, whereas VM patients display relatively symmetrical SIR values. **(C)** Shows the distribution of SIR values across MD ipsilateral ears, MD contralateral ears, and VM ipsilateral ears, revealing that SIR in MD ipsilateral ears is significantly higher than in both MD contralateral ears and VM ears. **(D)** The cochlear SIR asymmetry index (c-SIR AI) is compared between MD and VM groups, with the MD group demonstrating significantly greater asymmetry (17.24 ± 10.93 vs. 6.52 ± 3.74, *p* < 0.001). Panels E and F depict SIR distributions across different cochlear endolymphatic hydrops (cEH) grades in MD **(E)** and VM **(F)** patients. No statistically significant differences in SIR were found among the various cEH grades in either group.

On gadolinium-enhanced MRI, the SIR of the ipsilateral ears in MD patients was 1.39 ± 0.15, which was significantly higher than that of the contralateral ears in MD patients (1.17 ± 0.13) and ears in VM patients (1.18 ± 0.18) (*p* < 0.001). Furthermore, no significant difference in SIR was observed between the ipsilateral and contralateral ears in VM patients. Additionally, the c-SIR AI was significantly higher in MD patients compared to VM patients, measuring 17.24 ± 10.93 versus 6.52 ± 3.74, respectively (*p* < 0.001, Figures 2A–D).

In terms of EH, all ipsilateral ears in the MD group exhibited varying degrees of hydrops. The median cEH and vEH grades were Endolymphatic hydrops grades are reported as median (Q1, Q3) to accurately reflect their discrete and rank-ordered nature respectively. These grades were significantly higher than those observed in the contralateral ears of the MD group, where the cEH and vEH grades were 1 (0, 1) and 1 (0, 1), as well as in the VM ears, which exhibited cEH and vEH grades of 0 (0, 1) and 0 (0, 1) (all *p* < 0.001). The ipsilateral cEH in MD patients was notably higher than that in their contralateral ears and in VM patients, with a predominance of moderate-to-severe hydrops. Specifically, 26 (55.3%) of the MD ipsilateral ears had grade 2 cEH, while 21 (44.7%) had grade 1 cEH. In contrast, the MD contralateral ears exhibited significantly lower cEH grades, with 21 ears (44.7%) showing no hydrops (grade 0) and 26 ears (55.3%) showing grade 1 cEH. VM patients displayed the lowest degree of cEH, with 23 ears (71.9%) presenting no hydrops and 9 ears (28.1%) showing grade 1 cEH; no grade 2 cEH was observed in the VM group. For vEH, the grade and frequency distributions also differed significantly among MD ipsilateral ears, MD contralateral ears, and VM patients. Among the 47 MD ipsilateral ears, only 4 (8.5%) showed no hydrops (grade 0), while 28 (59.6%) exhibited grade 1 vEH, 12 (25.5%) had grade 2, and 3 (6.4%) had grade 3. In contrast, MD contralateral ears showed only 2 cases of grade 2 vEH, 24 cases (51.1%) of grade 1, and 21 cases (44.7%) of grade 0. VM patients exhibited milder vEH: among 32 patients, 19 (59.4%) had grade 0 vEH, 12 (37.5%) had grade 1, and only 2 (6.3%) had grade 2 vEH. No significant differences were observed between the VM group and MD contralateral ears regarding hearing thresholds, EH grades, or SIR values (*p* > 0.05 for all comparisons). The detailed distributions of EH grades are presented in Supplementary Table 1 (Figures 1A,C).

Among MD patients, no significant difference in the SIR was observed between ears with different grades of cEH (*p* = 0.065). The mean SIR was 1.34 ± 0.14 in ears with grade 1 cEH (n = 21) and 1.42 ± 0.15 in ears with grade 2 cEH (n = 26), indicating no statistically significant difference between the two groups. Similarly, in VM patients, the mean SIR was 1.20 ± 0.18 in ears without cEH (grade 0, n = 23) and 1.13 ± 0.16 in ears with grade 1 cEH (n = 9), with no significant difference observed between these groups (*p* = 0.342; see Figures 2E,F).

MD exhibited a distinct pattern of unilateral involvement on the ipsilateral side, evidenced by: (1) significantly higher PTA in the ipsilateral ears compared to those in the contralateral ears and VM patients; (2) significantly greater grades of cochlear and vestibular endolymphatic hydrops (cEH and vEH) in the ipsilateral ears; (3) a marked increase in blood-labyrinth barrier (BLB) permeability, as demonstrated by elevated SIR values in the ipsilateral ears; and (4) in contrast, the VM group displayed significantly lower c-SIR AI compared to MD, indicating a more symmetric pattern of cochlear involvement. Notably, some VM patients exhibited bilaterally elevated SIR values (Figures 1D, 2B), suggesting the potential presence of bilaterally increased BLB permeability as an underlying pathophysiological feature in VM (Table 2).

**Table 2:** Pure tone audiometry and imaging findings in patients with meniere’s disease and vestibular migraine.

Items	MD		VM
	Ipsilateral (*n* = 47)	contralateral (*n* = 47)	Ipsilateral (*n* = 32)
Pure tone audiometry test (dB HL)			
PTA	41.0 (33.8,59.0) ^ac^	23.0 (15.0,26.3) ^a^	16.3 (11.6,23.8) ^c^
Low Frequency	45.0 (37.5,52.5) ^ac^	15.0 (10,22.5) ^a^	12.5 (8.1,15.0) ^c^
Middle Frequency	42.5 (27.5,60.0) ^ac^	22.5 (12.5,25.0) ^a^	15.0 (10.0,24.4) ^c^
High Frequency	57.5 (47.5,67.5) ^ac^	35.0 (17.5,47.5) ^a^	31.3 (12.5,46.9) ^c^
Blood-labyrinth barrier permeability			
SIR	1.39 ± 0.15^bd^	1.17 ± 0.13 ^b^	1.18 ± 0.18 ^d^
c-SIR AI(%)	17.24 ± 10.93^d^		6.52 ± 3.74^d^
Endolymphatic hydrops			
Cochlear hydrops	2 (1,2) ^ac^	1(0,1) ^a^	0 (0,1) ^c^
None	0 (0.0%)	21 (44.7%)	23 (71.9%)
Grade I	21 (44.7%)	26 (55.3%)	9 (28.1%)
Grade II	26 (55.3%)	0 (0.0%)	0 (0.0%)
Grade ≥I	47 (100%) ^eg^	26 (55.3%)^g^	9 (28.1%) ^e^
Vestibular hydrops	1 (1,2) ^ac^	1 (0,1) ^a^	0 (0,1) ^c^
None	4 (8.5%)	21 (44.7%)	19 (59.4%)
Grade I	28 (59.6%)	24 (51.1%)	12 (37.5%)
Grade II	12 (25.5%)	2 (4.3%)	1 (3.1%)
Grade III	3 (6.4%)	0 (0.0%)	0 (0.0%)
Grade ≥I	43 (91.5%) ^eg^	24 (55.3%)^g^	13 (40.6%) ^e^
Grade ≥II	15 (31.9%)^fg^	2 (4.3%)^g^	1 (3.1%)^f^

^a^Wilcoxon signed-rank test, *p* < 0.001; ^b^Paired *t*-tests, *p* < 0.001; ^c^Mann–Whitney U-test, *p* < 0.001; ^d^Independent samples *t*-test, *p* < 0.001; ^e^Additional chi-square test (cEH ≥Grade I, *χ*^2^ = 43.2, *p* < 0.001; vEH ≥Grade I, *χ*^2^ = 22.31, *p* < 0.001); ^f^Fisher’s exact test (vEH ≥Grade II, *p* < 0.001); ^g^McNemar’s test (vEH ≥Grade I, *χ*^2^ = 6.13, *p* = 0.013; vEH ≥Grade I, *χ*^2^ = 8.47, *p* = 0.002; cEH≥ Grade I, *χ*^2^ = 21.00, *p* < 0.001), PTA, pure-tone average; SIR, signal intensity ratio; c-SIR AI, cochlear signal intensity ratio asymmetry index.

### 3.3 Imaging predictors of hearing loss in MD ipsilateral ears

**Figure 3:**
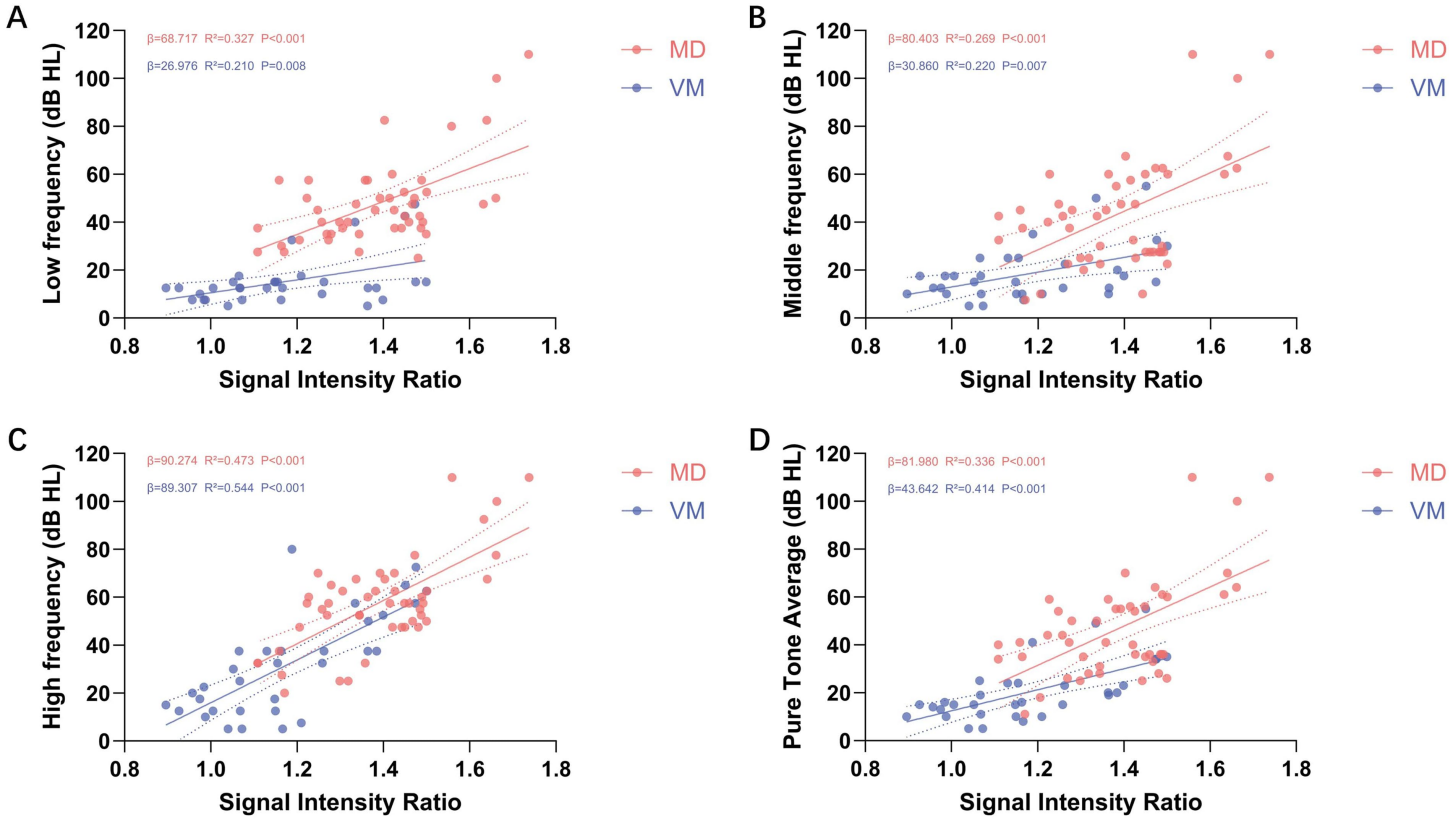
Illustrates the simple linear regression analysis between cochlear signal intensity ratio (SIR) and hearing thresholds across various frequencies. **(A–D)** Depict the relationships between SIR and low-frequency **(A)**, mid-frequency **(B)**, high-frequency **(C)**, and pure-tone average (PTA; **D**) thresholds, respectively. The analysis reveals a positive correlation between SIR and hearing thresholds across all frequency ranges (*β* > 0), with all associations achieving statistical significance (*p* < 0.01). Red dots represent the ipsilateral ears of patients with Meniere’s disease (MD), while blue dots indicate the ears of patients with vestibular migraine (VM). Each dot corresponds to the SIR value and hearing threshold of an individual ear at a specific frequency.

In the simple linear regression analysis of low-frequency hearing thresholds, SIR, cEH, and vEH emerged as significant predictors of low-frequency hearing loss (*p* < 0.05 for all). The regression coefficients (*β*) were 68.717 (95% CI: 39.143–98.291) for SIR, 22.624 (95% CI: 14.317–30.930) for cEH, and 13.000 (95% CI: 6.605–19.395) for vEH. The coefficients of determination (*R*^2^) were 0.327, 0.401, and 0.271, respectively, with cEH demonstrating the strongest explanatory power for low-frequency hearing loss. Subsequent multiple linear regression analysis indicated that SIR remained the strongest independent predictor after adjusting for other variables (*β* = 52.479, 95% CI: 31.181–73.778; standardized *β* = 0.437, *p* < 0.001), followed by cEH grade (*β* = 14.291, 95% CI: 9.189–29.471; standardized *β* = 0.419, *p* < 0.001). The overall model yielded an R^2^ of 0.692. No significant collinearity was detected between cEH and SIR, with all variance inflation factor (VIF) values being less than 5. Detailed results are presented in Table 3 and Figure 3.

**Table 3:** Imaging predictors of hearing loss in MD ipsilateral ears: simple and multiple linear regression models.

Items	Simple linear regression			Multiple linear regression				
	*β* (95% CI)	*p* value	*R* ^2^	*β* (95% CI)	Standardized *β*	*p* value	*R* ^2^	VIF
	Low Frequency (dB HL)				Low Frequency (dB HL)			
Age	0.273 (−0.085–0.631)	0.132	0.050					
Course	0.010 (−0.052–0.072)	0.749	0.002					
SIR	68.717 (39.143–98.291)	**0.000**	0.327	52.479 (31.181–73.778)	**0.437**	**0.000**	0.692	1.080
cEH	22.624 (14.317–30.930)	**0.000**	0.401	14.291 (7.629–20.953)	0.400	**0.000**	0.692	1.193
vEH	13.000 (6.605–19.395)	**0.000**	0.271	9.047 (4.559–13.536)	0.363	**0.000**	0.692	1.111
	Middle Frequency (dB HL)			Middle Frequency (dB HL)				
Age	0.525 (0.078–0.972)	**0.022**	0.110	0.071 (−0.288–0.431)	0.045	0.691	0.582	1.282
Course	0.041 (−0.039–0.120)	0.308	0.023					
SIR	80.403 (40.646–120.161)	**0.000**	0.269	58.003 (24.664–91.342)	0.374	**0.001**	0.582	1.142
cEH	28.251 (17.314–39.1188)	**0.000**	0.376	19.330 (9.189–29.471)	**0.419**	**0.000**	0.582	1.193
vEH	14.179 (5.503–22.854)	**0.002**	0.194	8.431 (1.027–15.835)	0.262	**0.027**	0.582	1.305
	High Frequency (dB HL)			High Frequency (dB HL)				
Age	0.493 (0.120–0.866)	**0.011**	0.136	0.149 (−0.160–0.459)	0.112	0.336	0.569	1.282
Course	0.035 (−0.032–0.102)	0.295	0.024					
SIR	90.274 (61.690–118.857)	**0.000**	0.473	80.139 (51.467–108.810)	**0.611**	**0.000**	0.569	1.142
cEH	14.707 (3.849–25.565)	**0.009**	0.142	5.084 (−3.638–13.805)	0.130	0.246	0.569	1.193
vEH	8.641 (0.849–16.378)	**0.030**	0.100	5.018 (−1.349–11.386)	0.184	0.119	0.569	1.305
	PTA (dB HL)			PTA (dB HL)				
Age	0.437 (0.024–0.849)	**0.039**	0.092	0.008 (−0.313–0.328)	0.005	0.962	0.603	1.282
Course	0.035 (−0.037–0.108)	0.330	0.021					
SIR	81.980 (47.335–116.606)	**0.000**	0.336	64.005 (34.334–93.676)	**0.452**	**0.000**	0.603	1.142
cEH	25.188 (15.058–35.318)	**0.000**	0.358	16.644 (7.618–25.669)	0.395	**0.001**	0.603	1.193
vEH	12.069 (4.020–20.117)	**0.004**	0.169	7.358 (0.769–13.948)	0.250	**0.030**	0.603	1.305

SIR, signal intensity ratio; cEH, cochlear endolymphatic hydrops; vEH, vestibular endolymphatic hydrops. Bold values indicate statistical significance (*p* ≤ 0.05); additionally, in the multiple linear regression analysis, the highest absolute standardized β value within each model is bolded.

In the simple linear regression analysis of mid-frequency hearing thresholds, age, SIR, cEH, and vEH emerged as significant predictors (*p* < 0.05 for all). The regression coefficients (*β*) along with their respective 95% confidence intervals (CI) were as follows: age, 0.525 (95% CI: 0.078–0.972); SIR, 80.403 (95% CI: 40.646–120.161); cEH, 28.251 (95% CI: 17.314–39.188); and vEH, 14.179 (95% CI: 5.503–22.854). The coefficients of determination (*R*^2^) for these predictors were 0.110, 0.269, 0.376, and 0.194, respectively, indicating that cEH had the greatest explanatory power for mid-frequency hearing loss. In the multiple linear regression analysis, cEH remained the strongest predictor after controlling for other variables (*β* = 19.330, 95% CI: 9.189–29.471; standardized *β* = 0.419, *p* < 0.001), followed by SIR (*β* = 58.003, 95% CI: 24.664–91.342; standardized *β* = 0.374, *p* < 0.001). The contribution of vEH was the least significant (*β* = 8.431, 95% CI: 1.027–15.835; standardized *β* = 0.262, *p* = 0.027). No significant collinearity was detected between cEH and SIR, as all variance inflation factor (VIF) values were below 5. Detailed results are illustrated in Figure 3 and Table 3.

In the simple linear regression analysis of high-frequency hearing thresholds, age, SIR, cEH, and vEH emerged as significant predictors of high-frequency hearing loss (*p* < 0.05 for all). The regression coefficients (*β*) along with their 95% confidence intervals (CI) were as follows: age: 0.493 (95% CI: 0.120–0.866), SIR: 90.274 (95% CI: 61.690–118.857), cEH: 14.707 (95% CI: 3.849–25.565), and vEH: 8.641 (95% CI: 0.849–16.378). The coefficients of determination (*R*^2^) for these predictors were 0.136, 0.473, 0.142, and 0.100, respectively, indicating that SIR possessed the strongest explanatory power regarding high-frequency hearing loss. In the multiple linear regression analysis, SIR remained the only significant independent predictor after adjusting for other variables (*β* = 80.139, 95% CI: 51.467–108.810; standardized *β* = 0.611, *p* < 0.001). Detailed results are illustrated in Figure 3 and Table 3.

The results of the simple linear regression analysis for PTA were consistent with those observed for mid-frequency hearing thresholds. Age, SIR, cEH, and vEH emerged as significant predictors of PTA, with all exhibiting *p*-values less than 0.05. The regression coefficients (*β*) and their corresponding 95% confidence intervals (CI) were as follows: age: 0.437 (95% CI: 0.024–0.849), SIR: 81.980 (95% CI: 47.335–116.606), cEH: 25.188 (95% CI: 15.058–35.318), and vEH: 12.069 (95% CI: 4.020–20.117). The coefficients of determination (R^2^) for these predictors were 0.092, 0.336, 0.358, and 0.169, respectively, indicating that cEH provided the most substantial explanatory power for PTA in the univariate analysis. However, in the multiple linear regression analysis, the predictive significance of age was no longer evident. SIR emerged as the strongest independent predictor of PTA, with a coefficient of *β* = 64.005 (95% CI: 34.334–93.676; standardized *β* = 0.452, *p* < 0.001), followed by cEH, which had a coefficient of *β* = 16.664 (95% CI: 7.618–25.669; standardized *β* = 0.414, *p* < 0.001). Detailed results are illustrated in Figure 3 and Table 3.

Disease duration was not a significant predictor of hearing thresholds at any frequency (*p* > 0.05). Although age was a significant predictor of PTA, mid-frequency, and high-frequency hearing thresholds in simple linear regression, it lost statistical significance after adjusting for EH and SIR in the multiple linear regression models (*p* = 0.962, 0.336, and 0.691, respectively). These findings indicate that neither age nor disease duration are major contributors to hearing loss in the ipsilateral ears of patients with Meniere’s disease (see Table 3).

### 3.4 Imaging predictors of hearing loss in VM ears

In patients with VM, the significant predictor of low-frequency hearing thresholds was SIR (*β* = 26.976, *R*^2^ = 0.210, *p* = 0.008). For mid-frequency thresholds and PTA, both age and SIR emerged as significant predictors (*p* < 0.05). Age had the most substantial impact on mid-frequency hearing thresholds (*β* = 0.303; standardized *β* = 0.400; *p* = 0.014), while SIR exhibited the strongest correlation with PTA (*β* = 70.807; standardized *β* = 0.593; *p* < 0.001). In terms of high-frequency thresholds, both age and SIR were significant predictors once again (*p* < 0.05), with SIR showing the highest explanatory power (*R*^2^ = 0.544), followed by age (*R*^2^ = 0.243). In the multiple linear regression model, after adjusting for age, SIR continued to be the strongest independent predictor of high-frequency hearing thresholds (*β* = 79.551; standardized *β* = 0.666; adjusted *R*^2^ = 0.659; *p* < 0.001). Detailed results are provided in Figure 3 and Table 4.

**Table 4:** Imaging predictors of hearing loss in VM ears: simple and multiple linear regression models.

Items	Simple linear regression			Multiple linear regression				
	*β* (95% CI)	*p* value	*R* ^2^	*β* (95% CI)	Standardized *β*	*p* value	*R* ^2^	VIF
	Low Frequency (dB HL)			Low Frequency (dB HL)				
Age	0.224 (−0.015–0.462)	0.065	0.109					
Course	−0.008 (−0.052–0.036)	0.703	0.005					
SIR	26.976 (7.456–46.496)	**0.008**	0.210					
cEH	−1.848 (−8.530–4.835)	0.576	0.011					
vEH	−2.974 (−9.801–3.851)	0.381	0.026					
	Middle Frequency (dB HL)			Middle Frequency (dB HL)				
Age	0.374 (0.127–0.620)	**0.004**	0.243	0.303 (3.735–44.762)	**0.400**	**0.014**	0.370	1.067
Course	0.016 (−0.033–0.065)	0.502	0.015					
SIR	30.860 (9.170–52.550)	**0.007**	0.220	24.249 (3.735–44.762)	0.368	**0.022**	0.370	1.067
cEH	−2.222 (−12.144–7.700)	0.651	0.007					
vEH	−3.861 (−11.456–3.737)	0.308	0.035					
	High Frequency (dB HL)			High Frequency (dB HL)				
Age	0.678 (0.231–1.124)	**0.004**	0.243	0.448 (0.132–0.764)	0.325	**0.007**	0.658	1.067
Course	0.000 (−0.090–0.089)	0.997	0.000					
SIR	89.307 (59.721–118.893)	**0.000**	0.544	79.551 (52.143–106.959)	**0.666**	**0.000**	0.658	1.067
cEH	−16.437 (−33.623–0.749)	0.060	0.113					
vEH	−3.560 (−17.526–10.405)	0.606	0.009					
	PTA (dB HL)			PTA (dB HL)				
Age	0.395 (0.143–0.646)	**0.003**	0.255	0.287 (0.079–0.495)	0.367	0.009	0.540	1.067
Course	0.008 (−0.042–0.059)	0.739	0.004					
SIR	43.642 (24.283–63.001)	**0.000**	0.414	37.395 (19.351–55.439)	**0.551**	**0.000**	0.540	1.067
cEH	−5.24 (−14.885–4.403)	0.276	0.039					
vEH	−4.778 (−12.541–2.984)	0.218	0.050					

SIR, signal intensity ratio; cEH, cochlear endolymphatic hydrops; vEH, vestibular endolymphatic hydrops. Bold values indicate statistical significance (*p* ≤ 0.05); ​additionally, in the multiple linear regression analysis, the highest absolute standardized β value within each model is bolded.

### 3.5 Discriminative value of imaging markers between Ménière’s disease and vestibular migraine

**Figure 4:**
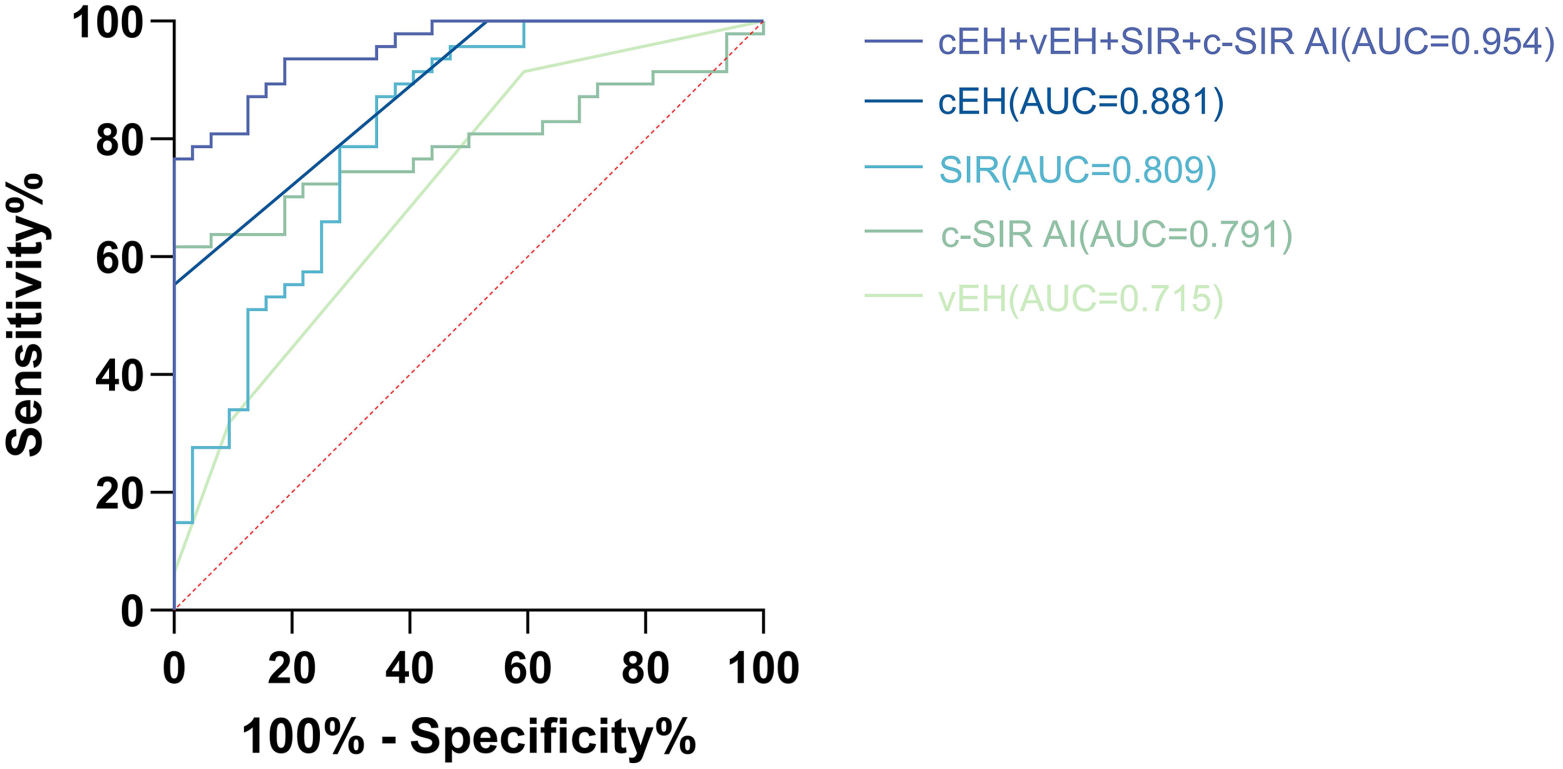
Illustrates the receiver operating characteristic (ROC) curve analysis of cEH, vEH, SIR, and c-SIR AI in distinguishing Ménière’s disease (MD) from vestibular migraine (VM). The optimal diagnostic thresholds identified were as follows: cEH at grade 2, vEH at grade 2, SIR greater than 1.22, and c-SIR AI exceeding 13.58%. At these specified cutoffs, the combined model demonstrated a sensitivity of 76.6% and a specificity of 100%, yielding an area under the curve (AUC) of 0.954 (95% CI, 0.915–0.993).

Logistic regression analysis indicated that MD was significantly associated with cEH, vEH, SIR, and c-SIR AI. Specifically, when cEH was graded ≥ 2, the sensitivity was 55.3%, specificity was 100%, and the area under the curve (AUC) was 0.881 (95% CI: 0.811–0.952). For vEH graded ≥ 2, the sensitivity was 31.9%, specificity was 90.6%, with an odds ratio (OR) of 4.028 (95% CI: 1.750–9.272), and an AUC of 0.715 (95% CI: 0.600–0.831). When SIR was ≥ 1.22, the sensitivity was 87.2%, specificity was 65.6%, and the AUC was 0.809 (95% CI: 0.708–0.910). The Z-score standardized OR was calculated to be 4.406 (95% CI: 2.222–8.738). For c-SIR AI, when it was ≥ 13.58%, the sensitivity was 61.7%, specificity was 100%, and the AUC was 0.791, with a Z-score standardized OR of 5.949 (95% CI: 2.425–14.593). When all four imaging criteria were combined, the model achieved a sensitivity of 76.6%, specificity of 100%, and an AUC of 0.954 (95% CI: 0.915–0.993; see Figure 4).

## 4 Discussion

In this study, we systematically evaluated the imaging and audiological characteristics of patients with MD and VM using 3D SPACE FLAIR MRI conducted 4 h after the intravenous administration of a gadolinium-based contrast agent. Our evaluation focused on EH, SIR, c-SIR AI, and frequency-specific hearing thresholds. The combined application of MRI-based parameters provides significant assistance in differentiating MD from VM in clinical settings. Furthermore, integrating imaging findings with audiological profiles may offer insights into the underlying pathophysiological mechanisms and support the development of tailored treatment strategies.

### 4.1 Imaging characteristics of the inner ear on gadolinium-enhanced MRI in Ménière’s disease vs. vestibular migraine

Our study demonstrates that delayed gadolinium-enhanced MRI of the inner ear provides objective imaging markers that assist in differentiating MD from VM. Specifically, the degree of EH was significantly greater in the MD group compared to the VM group. Patients with MD frequently exhibited marked cEH, whereas those with VM predominantly presented mild hydrops, with only a few cases showing severe EH (*p* < 0.001; see Table 2). Among the 47 patients diagnosed with MD, 15 exhibited vEH of grade 2 or higher, whereas only 2 out of the 32 patients with VM demonstrated grade 2 vEH. In the majority of these high-grade vEH cases, the hydrops predominantly involved the saccule, with the utricle being less frequently affected. This observed pattern is consistent with previous studies, which indicate that saccular hydrops generally precedes utricular involvement. This phenomenon may be attributed to differences in endolymphatic drainage mechanisms and the varying sensitivities of these structures to hydrodynamic pressure (8). Our findings are also consistent with previous reports indicating that the prevalence of EH in MD ranges from 80 to 100%, whereas in VM, EH has been reported in only 5 to 30% of patients (15, 16, 18, 19, 26). Cochlear EH is generally believed to correlate closely with hearing loss, particularly in the low- and mid-frequency ranges (6, 7, 10, 27). In contrast, the extent of vEH has not shown a significant correlation with vestibular test results or hearing thresholds (28). However, fluctuating EH may be associated with the waxing and waning of vertigo symptoms (29). Although the MD group exhibited higher rates of abnormal caloric and vHIT results compared to the VM group, these differences did not reach statistical significance (*p* = 0.182 and *p* = 0.566, respectively), which is inconsistent with some prior literature (3). This discrepancy suggests that conventional vestibular function tests may not fully capture the underlying pathophysiological distinctions between MD and VM. Variability in findings across studies may be attributed to differences in disease dynamics and methodological approaches. Vestibular evoked myogenic potentials (VEMPs), which assess otolith organ (saccule and utricle) function, were not analyzed in this study due to a high rate of missing data. This limitation stemmed from restricted availability of specialized equipment and the retrospective nature of some data collection. According to Valerie Kirsch, a neurovascular mechanism may underpin the VM phenotype, which typically presents with vestibular-predominant, low-grade, and bilaterally symmetric EH (16). In our study, among 32 VM ears, 12 (37.5%) exhibited grade 1 vEH, and only one ear (3.1%) presented with grade 2 vEH. Nine ears (28.1%) displayed grade 1 cEH. Notably, eight patients had symmetric grade 1 vEH in both ears, and six patients had bilateral grade 1 cEH, consistent with Kirsch’s hypothesis (see Supplementary Table 1).

The Signal Intensity Ratio (SIR), which quantifies perilymphatic enhancement, directly reflects the permeability of the Blood-Labyrinth Barrier (BLB). The BLB is formed by tight junctions between cochlear vascular endothelial cells and functions to separate inner ear fluids (endolymph and perilymph) from systemic circulation, thereby maintaining the unique ionic composition (e.g., high K^+^ and low Na^+^ in endolymph) and electrochemical potential within the cochlea. Pathological factors such as hypoxia, inflammation, and mechanical stress can compromise the integrity of the BLB, resulting in increased permeability (21). Our results indicated that the SIR of the ipsilateral ears in MD patients was significantly higher than that of VM patients (1.39 ± 0.15 vs. 1.18 ± 0.18, *p* < 0.001). Furthermore, nearly all MD ears exhibited higher SIR values than their contralateral ears (1.39 ± 0.15 vs. 1.17 ± 0.13, *p* < 0.001), confirming the prominent and unilateral involvement of BLB dysfunction in MD (15, 19, 20, 22, 24). Endolymphatic hydrops may contribute to increased BLB permeability by exerting mechanical compression on microvascular-rich cochlear structures, such as the stria vascularis and spiral ligament vessels. Additionally, localized inflammation and ionic dysregulation, particularly potassium accumulation, may activate endothelial damage pathways, further exacerbating permeability and resulting in the characteristic unilateral gadolinium enhancement pattern observed in MD. To address the limitations of subjective visual evaluation of perilymphatic enhancement, we introduced a quantitative index—the cochlear SIR asymmetry index (c-SIR AI)—to objectively assess interaural differences in BLB permeability. The c-SIR AI was significantly higher in MD patients compared to those with VM (17.24 ± 10.93% vs. 6.52 ± 3.74%, *p* < 0.001), highlighting the diagnostic value of BLB asymmetry. Importantly, the lower c-SIR AI in VM patients does not indicate the absence of BLB disruption; rather, it suggests a potentially distinct pathophysiological mechanism. VM may involve bilateral, relatively symmetric BLB dysfunction due to widespread neurovascular coupling disturbances. This bilateral pattern aligns with Valerie Kirsch’s hypothesis that VM primarily affects both ears in a symmetric and vestibular-dominant manner (16).

### 4.2 Relationship between blood–labyrinth barrier permeability, endolymphatic hydrops, and hearing loss

This study revealed that both increased BLB permeability and the degree of cEH are associated with hearing loss in patients with MD. However, these two pathological factors seem to affect different frequency ranges and are not significantly correlated with one another. This suggests that two mechanisms may contribute to hearing impairment in MD.

On one hand, the extent of cEH predominantly affects low- and mid-frequency hearing thresholds. Our findings indicate that greater degrees of cochlear hydrops are associated with higher thresholds at low frequencies in the ipsilateral ears of patients with MD. This observation aligns with the classic audiological profile of MD, which is typically characterized by fluctuating low- to mid-frequency sensorineural hearing loss during the early stages (18, 26, 30). Pathophysiologically, endolymphatic hydrops tends to accumulate initially in the apical turn of the cochlea, where low-frequency processing occurs. The resulting mechanical distortion of the basilar membrane in this region is believed to impair hair cell function and disrupt cochlear signal transduction (8).

On the other hand, elevated SIR—reflecting greater disruption of the BLB—was positively correlated with hearing loss across all frequency ranges, with the strongest association observed in the high-frequency domain. Damage to the BLB may directly impair cochlear hair cell function. Increased permeability allows inflammatory mediators and plasma components to leak into the perilymph, potentially exerting cytotoxic effects on sensory cells. Hair cells in the basal turn of the cochlea, which are responsible for high-frequency sound processing, have higher metabolic demands and are therefore more vulnerable to such insults. This frequency-specific vulnerability is further supported by our findings in VM patients, in whom SIR emerged as a significant predictor of hearing thresholds, particularly at high frequencies. Even after adjusting for age, SIR remained the strongest independent predictor of high-frequency hearing loss (standardized *β* = 0.666, R^2^ = 0.658, *p* < 0.001). This observation aligns with the proposed neurovascular pathophysiology of VM, wherein recurrent transient inner ear ischemia may lead to subtle BLB disruption (16). The resulting damage is typically bilateral and symmetric, consistent with the slowly progressive and symmetrical hearing decline commonly observed in VM patients (3, 17). Similarly, Li reported a linear association between SIR and both low- and high-frequency hearing thresholds, noting that these relationships remained significant after age adjustment (9). However, due to multicollinearity among frequency bands, the study did not further delineate the relative strengths of these associations.

In our study, the SIR values did not significantly differ across various grades of EH in the ipsilateral ears. However, this finding does not provide sufficient evidence to conclude that SIR and EH represent entirely independent pathological processes. The observed lack of correlation may be attributed to current methodological limitations, including inadequatedly refined EH grading systems and the absence of three-dimensional SIR quantification techniques. Notably, in patients with MD, after accounting for the effect of endolymphatic hydrops (cEH/vEH), the predictive contribution of SIR to low-frequency hearing loss increased. A combined model incorporating both SIR and EH accounted for a larger proportion of variance in low-frequency hearing thresholds (R^2^ = 0.699). Furthermore, diagnostic performance was optimized when multiple imaging markers were integrated (AUC = 0.954), suggesting that SIR may function as an upstream pathological factor. It may impair hearing either directly, by increasing permeability and disrupting oxygen homeostasis in hair cells—or indirectly by contributing to the development of EH. The relationship between SIR and EH in MD remains controversial (9, 18, 20, 31). Differences in patient inclusion criteria, imaging protocols, and MRI sequence parameters may account for discrepancies across studies. For instance, Zhang (31) and Li (9) calculated SIR based on randomly selected cochlear basal slices, which may have introduced systematic errors. de Pont (18) included patients with any MRI-detected EH, including those with potential VM, possibly conflating the association between SIR and EH. Additionally, perilymphatic enhancement is also observed in other acute vestibular syndromes such as sudden sensorineural hearing loss and acute unilateral vestibulopathy (20, 32, 33), supporting its role as a sensitive imaging biomarker of BLB dysfunction, rather than being specific to MD alone.

### 4.3 Clinical implications

Although EH has long been regarded as a hallmark of MD, it is not exclusive to this condition. A subset of patients of VM also exhibit varying degrees of EH, which limits the diagnostic specificity of EH alone. In our study, the diagnostic performance of cochlear EH (AUC = 0.881) and vestibular EH (AUC = 0.694) was suboptimal when considered as single predictors. However, the combination of multiple objective imaging parameters derived from delayed gadolinium-enhanced inner ear MRI significantly enhanced diagnostic accuracy. When the following four criteria were simultaneously satisfied—cochlear EH grade ≥ 2, vestibular EH grade ≥ 2, SIR ≥ 1.22, and cochlear SIR asymmetry index (c-SIR AI) ≥ 13.58%—the specificity for diagnosing MD reached 100%, with a sensitivity of 76.6%, and an area under the ROC curve (AUC) as high as 0.954, indicating excellent discriminatory power. For patients with suspected MD who present with overlapping migraine features or atypical clinical symptoms, early use of gadolinium-enhanced MRI is strongly recommended. This approach may help reduce misdiagnosis or underdiagnosis, facilitating timely and targeted therapeutic intervention.

Despite our exclusion of patients with concurrent MD and VM diagnoses, a considerable subset of the cohort still exhibited overlapping clinical features. Among the patients diagnosed with MD, 8 out of 47 (17%) had a history of migraine. Additionally, 4 out of 32 patients diagnosed with VM exhibited symptoms similar to those of MD. However, these patients did not strictly meet the diagnostic criteria for either MD or VM (1, 2), and therefore do not fulfill the criteria for comorbidity between VM and MD (1, 2). This highlights that MD and VM are not entirely distinct entities, but rather may share pathophysiological pathways, leading to symptom overlap. In our study, for instance, some VM patients demonstrated mild bilateral endolymphatic hydrops on MRI accompanied by bilaterally elevated SIR values. These findings suggest the presence of diffuse BLB dysfunction in VM. BLB impairment may concurrently trigger disturbances in inner ear ionic homeostasis and inflammatory responses, leading to the development of EH and hair cell damage. This, in turn, can result in MD-like inner ear changes accompanied by VM-like episodes. Such a mechanism provides a plausible explanation for the overlapping spectrum between MD and VM, and increased BLB permeability may represent a shared pathophysiological pathway linking the two disorders. However, our team currently adopts a cautious interpretation of this phenomenon, as the present study provides only indirect evidence and the number of patients with overlapping symptoms was limited. Comprehensive evaluation of patients with overlapping symptoms—including audiometric assessment, BLB permeability, and EH quantification—together with longitudinal follow-up, may yield more robust evidence in the future.

In summary, the significance of this article primarily lies in its exploration of how to integrate imaging findings to guide individualized treatment. First, the degree of perilymphatic enhancement has been shown to correlate positively with both the duration of clinical symptoms and hearing thresholds in patients with MD (9), suggesting that a simple visual comparison of ipsilateral and contralateral ears is insufficient for personalized clinical decision-making. Notably, previous studies have demonstrated that the SIR in the contralateral ear of MD patients is higher than that of healthy controls or patients with sudden sensorineural hearing loss (20, 22). Given that 20–50% of unilateral MD cases may eventually progress to bilateral involvement (34, 35), elevated SIR in the asymptomatic ear may reflect early BLB dysfunction and serve as a harbinger of disease progression. In such cases, early intervention aimed at improving inner ear microcirculation and reducing inflammation could potentially delay or prevent contralateral involvement. Second, in patients with VM, symmetric bilateral perilymphatic enhancement may indicate diffuse and severe BLB disruption, which is often associated with more prominent cochlear symptoms. Careful differentiation from MD is essential in these cases, as MD more typically presents with marked unilateral enhancement. These two scenarios may represent a cross-subtype within the MD–VM disease spectrum. Restoration of microvascular homeostasis and management of the underlying neurovascular triggers should be prioritized. Conversely, MD patients with significant EH but without marked BLB disruption may represent a subtype driven predominantly by structural impairment of endolymphatic circulation. These individuals may respond positively to diuretics or surgical intervention, indicating a distinct therapeutic subgroup. Furthermore, the coexistence of high-frequency hearing loss and elevated SIR may imply ongoing or irreversible cochlear injury. Serial monitoring of these parameters could serve as a potential biomarker for treatment response.

## 5 Limitations

This study presents several limitations. First, all participants were recruited from a single specialized center, resulting in a relatively high proportion of middle- to late-stage MD cases, which may have introduced selection bias. Second, the Bernaerts grading system relies on semi-quantitative visual assessment and may be subject to inter-observer variability. Third, the absence of healthy control subjects in this study somewhat limits the interpretability of our findings. Future research will address this limitation by including a cohort of healthy volunteers, which will allow us to further validate the characteristic features of gadolinium-enhanced MRI of the inner ear in the general population as a comparative baseline. Additionally, the SIR was measured from a single slice at the cochlear basal turn, which may not fully capture the spatial extent of inner ear damage. Finally, the causal relationship between EH and SIR remains to be validated through longitudinal studies. Future research should involve larger, multicenter cohorts, AI-assisted quantitative evaluation, and multimodal data integration to enhance the generalizability and mechanistic interpretation of the findings.

## 6 Conclusion

Delayed gadolinium-enhanced MRI of the inner ear can assist in the clinical differentiation between VM and MD. The combination of endolymphatic hydrops, signal intensity ratio, and cochlear SIR asymmetry index exhibited excellent diagnostic performance. Notably, cochlear endolymphatic hydrops predominantly contributes to low- and mid-frequency hearing loss, while an elevated signal intensity ratio is independently associated with high-frequency hearing impairment. By integrating imaging and audiological profiles, clinicians can accurately characterize cochlear pathology, enabling the development of tailored treatment strategies for affected patients.

## Data Availability

The raw data supporting the conclusions of this article will be made available by the authors, without undue reservation.
